# Hexokinase is necessary for glucose-mediated photosynthesis repression and lipid accumulation in a green alga

**DOI:** 10.1038/s42003-019-0577-1

**Published:** 2019-09-19

**Authors:** Melissa S. Roth, Daniel J. Westcott, Masakazu Iwai, Krishna K. Niyogi

**Affiliations:** 1 Howard Hughes Medical Institute, Department of Plant and Microbial Biology, University of California, Berkeley, CA 94720-3102 USA; 2 Molecular Biophysics and Integrated Bioimaging Division, Lawrence Berkeley National Laboratory, Berkeley, CA 94720 USA

**Keywords:** Photosynthesis, Biodiesel, Cellular microbiology, Plant physiology, Plant molecular biology

## Abstract

Global primary production is driven largely by oxygenic photosynthesis, with algae as major contributors. The green alga *Chromochloris zofingiensis* reversibly switches off photosynthesis in the presence of glucose in the light and augments production of biofuel precursors (triacylglycerols) and the high-value antioxidant astaxanthin. Here we used forward genetics to reveal that this photosynthetic and metabolic switch is mediated by the glycolytic enzyme hexokinase (CzHXK1). In contrast to wild-type, glucose-treated *hxk1* mutants do not shut off photosynthesis or accumulate astaxanthin, triacylglycerols, or cytoplasmic lipid droplets. We show that CzHXK1 is critical for the regulation of genes related to photosynthesis, ketocarotenoid synthesis and fatty acid biosynthesis. Sugars play fundamental regulatory roles in gene expression, physiology, metabolism, and growth in plants and animals, and we introduce a relatively simple, emerging model system to investigate conserved eukaryotic sugar sensing and signaling at the base of the green lineage.

## Abstract (web summary)

In plants, exogenous glucose represses photosynthesis but this phenomenon has been little studied in green algae. Roth et. al. discover through a forward genetics screen in *C. zofingiensis* that hexokinase 1 is needed to turn off photosynthesis in the presence of glucose and for glucose-mediated upregulation of lipid metabolism.

## Introduction

Global net primary production (NPP) depends on regulation of oxygenic photosynthesis and primary carbon metabolism. Algae and cyanobacteria are responsible for approximately half of global NPP^1^. Algae can have flexible metabolism, and they can grow either photoautotrophically or heterotrophically with an exogenous reduced carbon source^2,3^. Many algae, including the reference alga *Chlamydomonas reinhardtii*, which can grow on a reduced carbon source, will also maintain photosynthesis and are thus capable of mixotrophic growth in the light^4^. In contrast, *Chromochloris zofingiensis*, a unicellular coccoid green alga that is closely related to *C. reinhardtii*^5^, reversibly turns off photosynthesis, degrades the photosynthetic apparatus, and reduces thylakoid membranes in the presence of glucose in the light^6^. *C. zofingiensis* can be cultivated on a variety of sugars and in multiple trophic modes, and it can produce high amounts of biofuel precursors and astaxanthin under conditions such as heterotrophy, nitrogen deprivation, and high light^5–9^. Recent commercial interest in this alga has grown due to its economically promising attributes^10^. The easily controlled, glucose-induced photosynthetic switch in *C. zofingiensis* offers the opportunity to gain insight into the molecular players and mechanisms regulating photosynthesis and carbon metabolism in algae, which will enable rerouting and engineering of metabolism to improve commercial prospects of algal bioproducts.

Sugars are main carbon and energy sources of cells. In many organisms, sugars have regulatory roles that affect metabolism, growth, aging, and stress resistance. In plants, sugars are key metabolic and regulatory molecules, and their roles in signaling have been extensively investigated^11,12^. The preferred carbon source for many organisms is glucose. Exogenous glucose has been shown to repress photosynthesis in plants while modulating growth and development^11,12^. However, a knowledge of the molecular players and mechanistic understanding of glucose signaling in green algae, a sister group to land plants, is presently lacking, likely due to the inability of *C. reinhardtii* to grow on glucose as a sole carbon source^13^. Identification of the molecular players and mechanisms underlying photosynthetic and metabolic changes in response to glucose and other sugars in green algae will provide a phylogenetic perspective on sugar signaling and reveal insights into fundamental mechanisms present at the base of the green lineage.

In this study, we investigated the regulation of photosynthesis and metabolism in *C. zofingiensis* in response to exogenous glucose, building on the foundation provided by the recent high-quality, chromosome-level genome and transcriptome of *C. zofingiensis*^5^. Through a forward genetics screen, we uncovered hexokinase1 (CzHXK1) as a master regulator of photosynthesis, carbon metabolism, and ketocarotenoid (astaxanthin) biosynthesis in *C. zofingiensis*. The *hxk1* mutants are deficient in turning off photosynthesis and accumulating astaxanthin and lipids in the presence of glucose. HXK, a key enzyme in carbon metabolism, generates glucose-6-phosphate (G6P) that can be used in a variety of pathways including glycolysis, the oxidative pentose phosphate pathway, and starch and cell wall biosynthesis^14^. HXK is also known to be an evolutionarily conserved eukaryotic glucose sensor in yeast and plants^11,12^. In contrast to yeast and plants, *C. zofingiensis* has a single gene encoding hexokinase^5^. Thus, our work establishes *C. zofingiensis* as a relatively simple system to investigate conserved mechanisms of sugar sensing and signaling in the green lineage.

## Results

### Genetics identifies hexokinase as a regulator of photosynthesis

To uncover the molecular players involved in the glucose-dependent photosynthetic switch in *C. zofingiensis*, we used forward genetics to identify mutants that maintain photosynthesis in the presence of glucose. Using UV mutagenesis followed by selection on the glucose analog, 2-deoxy-d-glucose (2-DOG), we generated eight independent mutant strains that did not shut off photosynthesis and grew in the presence of 2-DOG with light. 2-DOG is transported into the cell and phosphorylated but cannot be metabolized by glycolysis^15–17^. 2-DOG has been utilized to investigate sugar sensing in a variety of organisms^15,16^. Wild-type (WT) *C. zofingiensis* cells sense the presence of 2-DOG and repress photosynthesis, resulting in cell death because 2-DOG is phosphorylated to a dead-end metabolic product. Mutants that are resistant to 2-DOG survive, because they do not shut off photosynthesis. To eliminate glucose transport mutants, we tested the growth of mutants in the dark. All eight mutants were able to grow in the dark on glucose, providing evidence that they can still take up and metabolize glucose. A detailed experiment on WT and two mutants showed that growth on glucose in the light is slightly faster than growth on glucose in the dark (Supplementary Fig. 1).

**Fig. 1:**
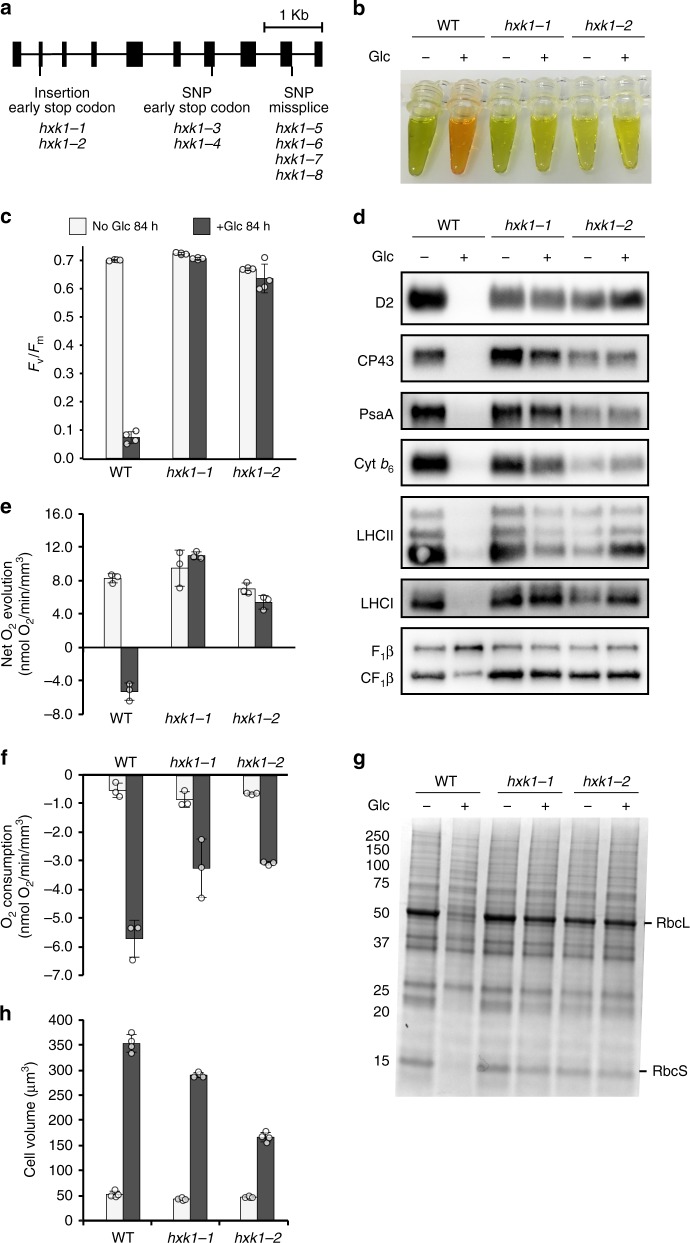
Mutants reveal hexokinase1 as regulator of photosynthesis. **a** Gene structure of *CzHXK1* with mutation location and type of *hxk1* mutations as determined by whole-genome sequencing (see Methods). **b** Image of extracted pigments from representative samples of WT and *hxk1* mutants after 84 h with and without glucose (Glc). **c** Maximum photosystem II efficiency (*F*_v_/*F*_m_) of *hxk1* mutants is insensitive to glucose. Data represent means ± SD (*n* = 4 biological replicates, individual data points shown). **d** Photosynthetic apparatus of *hxk1* mutants is insensitive to glucose. Immunoblot analysis of PSII (D2, CP43), PSI (PsaA), cytochrome *b*_6_*f* (Cyt *b*_6_), light-harvesting complex of PSII (LHCII), light-harvesting complex of PSI (LHCI), and ATP synthase (AtpB) subunits after 84 h with and without glucose. The global AtpB antibody detected both the chloroplastic AtpB at ~50 kDa and the mitochondrial AtpB at ~70 kDa. Samples were normalized to total protein, and 10 µg of protein were loaded in each well. See Fig. 1g for stained total protein gel loading control. Images of the detected chemiluminescent signal on the complete membranes are available in Supplementary Fig. 4. **e** Net oxygen evolution (oxygen production in the light, 100 μmol photons m^−2^ s^−1^) of *hxk1* mutants is insensitive to glucose. Data represent means ± SD (*n* = 3 biological replicates, individual data points shown). **f** Respiration (oxygen consumption in the dark) of *hxk1* mutants is sensitive to glucose. Data represent means ± SD (*n* = 3 biological replicates, individual data points shown). **g** SDS-PAGE analysis of total protein extracted from whole cells and stained with Coomassie brilliant blue. Samples were normalized to total protein, and 10 µg of protein were loaded in each well. **h** Cell volume of *hxk1* mutants increases with glucose. Data represent means ± SD (*n* = 4 biological replicates, individual data points shown)

Next, we used a whole-genome sequencing pipeline to identify potentially causative mutations^18,19^. High-coverage genome sequencing (average 94x coverage) revealed that all eight mutants had disruptive mutations in the single gene encoding hexokinase (*CzHXK1*) (Fig. 1a, Supplementary Table 1). These mutations were confirmed by Sanger sequencing. HXK1 is both a sugar kinase and a well-studied glucose sensor and regulator in plants and yeast^12,20,21^. Three classes of *hxk1* mutants were found: class 1 with an insertion (C471CG) that causes an early stop codon in the second exon; class 2 with a base pair change (C3426T) that causes an early stop codon in exon 7; and class 3 mutants with a T4795G mutation that disrupts a splice site between exons 9 and 10 (Supplementary Table 1). Each of the eight mutants represents an independently generated allele, as verified by analysis of the number and locations of non-causative mutations, including SNPs and INDELs (Supplementary Table 1). The ability of the *hxk1* mutants to grow in the dark on glucose is likely due to the presence of a glucokinase gene (Cz06g03010) in the genome^5^.

### Hexokinase is required for the photosynthetic switch

To characterize the response of *hxk1* mutants to glucose, we added glucose to photoautotrophic cultures of *hxk1* mutants and WT and conducted various physiological analyses after 84 h, when WT exhibits a complete shut-off of photosynthesis^6^. For these experiments, we used two independent class 1 mutants, *hxk1-1* and *hxk1-2*. These strains share an identical mutation that produces an early stop codon in *CzHXK1*, and thus they are most likely null mutations affecting HXK activity, but they contain different background mutations in their genomes that resulted from UV mutagenesis (Supplementary Table 1). Therefore, the shared phenotypes can be attributed specifically to the shared mutation in *CzHXK1* and are not likely explained by UV-induced mutations in other genes.

In response to glucose, WT changed color dramatically from green to orange, while *hxk1* mutants with and without glucose remained similar in color (Fig. 1b). The *hxk1* mutants did not show a decrease in photosynthesis with glucose (Fig. 1c–g). In contrast to WT, which exhibited a severe decline of photosystem (PS) II efficiency at 84 h, *F*_v_/*F*_m_ of *hxk1-1* and *hxk1-2* did not decline with glucose (Fig. 1c). Furthermore, *hxk1* mutants continued to evolve oxygen in the light at comparable rates in the presence and absence of glucose, whereas WT decreased oxygen evolution to the point of net oxygen consumption in the light (Fig. 1e). Results were normalized to cell volume, because both WT and *hxk1* cells grew larger with glucose (Fig. 1h). As the glucose-treated cells grew larger in volume, their rate of cell division decreased (Supplementary Fig. 1)^6^. Our previous study showed that the decreases in PSII efficiency and oxygen evolution in the light in WT are reversible with the removal of glucose within 24 h^6^. Respiration (oxygen uptake in the dark) with glucose increased in both WT and *hxk1* cells (Fig. 1f). The PSII efficiency and oxygen evolution data suggest that CzHXK1 is necessary for glucose to shut off photosynthesis.

We used immunoblot analysis to characterize the changes in abundance of protein subunits of the photosynthetic apparatus. In WT, subunits of PSII (D2 and CP43), PSI (PsaA), cytochrome *b*_6_*f* complex (Cyt *b*_6_), and light-harvesting complexes (LHCI and LHCII) decreased substantially with glucose (Fig. 1d). This glucose-induced loss of the photosynthetic apparatus is reversible with the removal of glucose within 24 h^6^. Chloroplastic and mitochondrial AtpB subunits (CF_1_β and F_1_β, respectively) were present in WT with and without glucose. With glucose, there was potentially a slight increase in the ratio of mitochondrial to chloroplastic AtpB, which is consistent with the increase in respiration. In contrast, immunoblot analysis of the *hxk1* mutants revealed that PSI, PSII, LHCs, Cyt *b*_6_*f* complex, and ATP synthase were relatively insensitive to glucose and showed no consistent increases or decreases with and without glucose (Fig. 1d). The loading control, an SDS-PAGE analysis of total protein extracts stained with Coomassie brilliant blue, also showed a dramatic decrease in the large and small subunits of the CO_2_-fixing enzyme Rubisco at 53 kDa and 15 kDa, respectively, in WT with glucose (Fig. 1g). These data suggest that the loss of the photosynthetic apparatus with glucose requires CzHXK1.

**Fig. 2:**
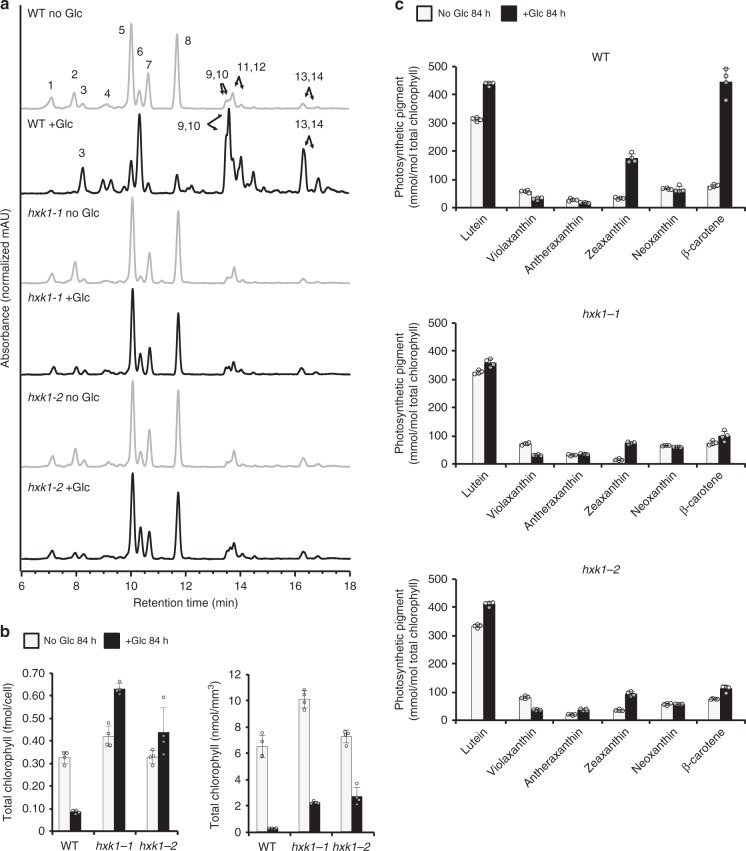
The pigment response of hexokinase1 mutants to glucose is attenuated. **a** Representative HPLC chromatograms of WT and *hxk1* mutants after 84 h with (black lines) and without (gray lines) glucose (Glc). 1, neoxanthin; 2, violaxanthin; 3, free astaxanthin; 4, antheraxanthin; 5, lutein; 6, zeaxanthin; 7, chlorophyll *b*; 8, chlorophyll *a*; 9 and 10, esterified astaxanthin; 11 and 12, β-carotene; 13 and 14, esterified astaxanthin. **b** Total chlorophyll (chlorophyll *a* and chlorophyll *b*) of WT and *hxk1* mutants after 84 h with and without glucose. Left graph is normalized per cell and right graph is normalized by cell volume. Data represent means ± SD (*n* = 4 biological replicates, individual data points shown). **c** Pigments relative to total chlorophyll of WT (top), *hxk1-1* (middle), and *hxk1-2* (bottom) after 84 h with and without glucose. Data represent means ± SD (*n* = 4 biological replicates, individual data points shown)

**Fig. 3:**
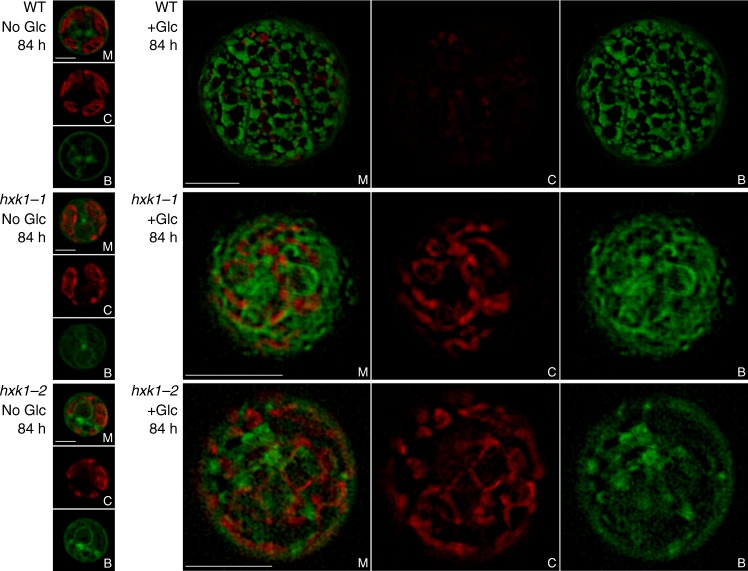
Hexokinase1 mutants are deficient in lipid droplet accumulation. Representative images of live-cell super-resolution structured illumination microscopy (SIM) of WT and *hxk1* mutants with and without glucose (Glc) showing (M) merged chlorophyll autofluorescence (650–730 nm) and BODIPY fluorescence (505–550 nm), (C) individual channel showing chlorophyll autofluorescence, and (B) individual channel showing BODIPY fluorescence. Scale bars, 2 µm for No Glc and 5 µm for +Glc

Levels of total chlorophyll (chlorophylls *a* and *b*) per cell decreased in WT but increased in the *hxk1* mutants with glucose (Fig. 2a, b). However, because the cells grew larger with glucose (Fig. 1h), the abundance of chlorophyll per cell volume decreased in both WT and the mutants (Fig. 2b). Live-cell imaging using super-resolution structured illumination microscopy (SIM) revealed a dramatic decrease in chlorophyll fluorescence intensity in WT with glucose, whereas chlorophyll fluorescence intensity remained comparable with and without glucose in *hxk1* mutants (Fig. 3). Altogether, measurements of photosynthesis and the photosynthetic apparatus provide evidence that HXK1 is necessary for the glucose-induced photosynthetic switch.

### Hexokinase is required for high astaxanthin accumulation

Under specific conditions, *C. zofingiensis* is well known to accumulate high amounts of astaxanthin^5–9^. Astaxanthin accumulates in esterified forms, first as fatty acyl mono-esters and secondarily as di-esters^22^. In WT with glucose, the astaxanthin mono- and di-esters accumulated as the culture changed from green to orange (Figs. 1b, 2a). In contrast, the *hxk1* mutants remained green with glucose, and we did not observe an increase in astaxanthin (Figs. 1b, 2a). Similar to WT, *hxk1* mutants did show an increase in the ratio of zeaxanthin to violaxanthin and an increase in β-carotene with glucose (Fig. 2d–f). Zeaxanthin and/or β-carotene are hypothesized to be precursors of astaxanthin in *C.zofingiensis*^5,10^. These data support that CzHXK1 is critical for the glucose-dependent accumulation of large amounts of astaxanthin.

### Hexokinase is required for accumulation of cytoplasmic lipid droplets

Neutral lipid staining of live cells followed by SIM provided insight into the formation of lipid droplets in the presence of glucose. In WT, a network of cytoplasmic lipid droplets accumulated near the cell membrane in glucose-treated cells (z-stack 1 in WT+glc 84 h, Fig. 3), which is consistent with previous work^6,23^. However, the cytoplasmic lipid droplets observed in WT were missing in *hxk1* mutants (Fig. 3). Instead of clearly defined lipid droplets as in WT, the lipid staining in *hxk1* mutants was diffuse. Overall, these data suggest that CzHXK1 is essential for the accumulation of cytoplasmic lipid droplets near the plasma membrane.

### Hexokinase is required for accumulation of TAGs

**Fig. 4:**
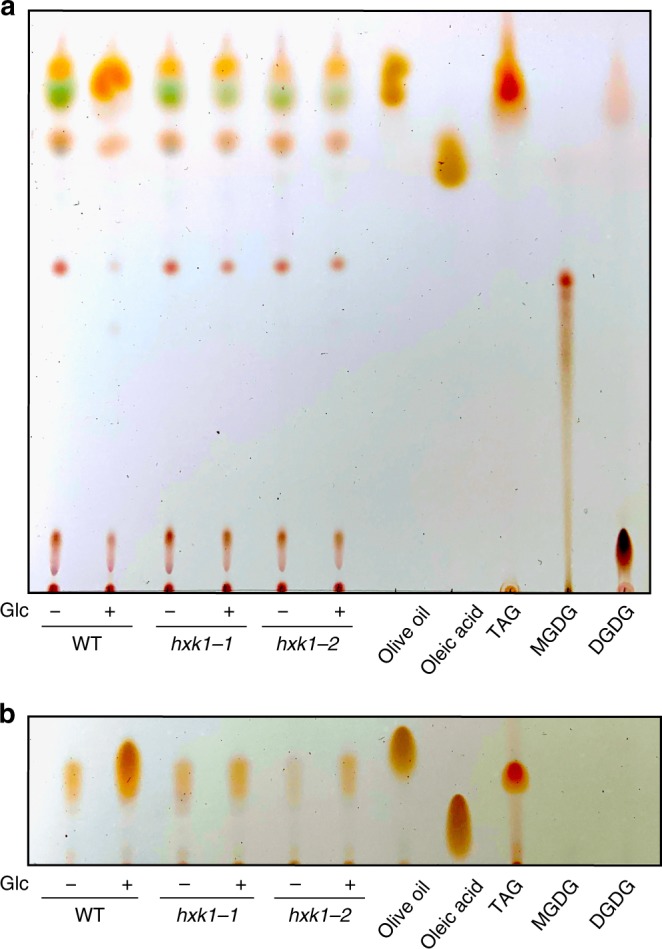
Hexokinase1 mutants are deficient in triacylglycerol accumulation. Lipid extracts of WT and *hxk1* mutants after 84 h with and without glucose (Glc) developed on silica TLC plates with **a** acetone:toluene:water (91:30:8) and **b** hexane:diethyl ether:glacial acetic acid (91:39:1.3). Solvent used in **a** shows more lipid classes and solvent used in **b** shows greater separation of TAG. Samples were normalized to culture biomass. 125 µg oleic acid, 50 µg TAG, 50 µg MGDG, 40 µg DGDG, and 2.5 µL of olive oil extract were loaded as standards

Commercial interest in *C. zofingiensis* has grown recently because it can accumulate high amounts of the preferred lipid precursor for biofuels, triacylglycerol (TAG), under nitrogen deprivation or with glucose^6–9^. Lipid content assayed using thin-layer chromatography showed that while WT accumulates high levels of TAG with glucose, the *hxk1* mutants do not accumulate TAG with glucose (Fig. 4). It is likely that in WT, TAG accumulates in the neutral cytoplasmic lipid droplets near the plasma membrane as visualized by SIM (Fig. 3). In addition, WT with glucose showed a reduction in the most abundant thylakoid lipids, monogalactosyldiacylglycerol (MGDG) and digalactosyldiacylglycerol (DGDG), as previously observed^6^. In contrast, the *hxk1* mutants did not decrease thylakoid lipids with glucose, and this result is consistent with the maintenance of the photosynthetic apparatus (Fig. 1). Taken together, these data provide evidence that CzHXK1 is important for TAG accumulation with glucose.

### Hexokinase regulates photosynthetic and metabolic gene expression

**Fig. 5:**
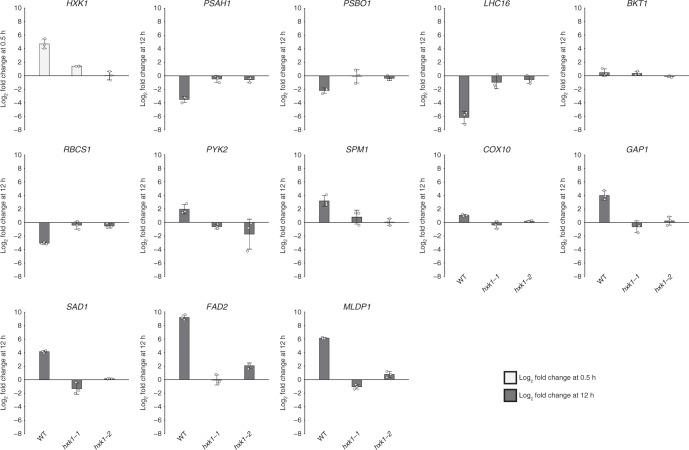
Mutants reveal CzHXK1-dependent transcriptional changes. qRT-PCR analysis of mRNA levels of select photosynthetic and metabolic genes (identified by RNA-Seq^6^) in WT and *hxk1* mutants. The log_2_-transformed fold change of mRNA level with glucose relative to a time-matched control without glucose at 0.5 h for *CzHXK1* and at 12 h for all other genes. Additional time points and genes are shown in Supplementary Fig. 2. Data represent means ± SD (*n* = 3 biological replicates, individual data points shown). Raw ΔC_T_ data and biological replicates are shown in Supplementary Fig. 3

To assess the role of CzHXK1-dependent gene expression in the glucose-dependent photosynthetic and metabolic switch, we measured changes in RNA abundance upon glucose addition to *hxk1* mutants as compared to WT using quantitative reverse transcription-PCR (qRT-PCR). We selected photosynthetic and metabolic genes identified by RNA-Seq^6^ and investigated RNA abundance fold changes with and without glucose at two time points after adding glucose, 0.5 h and 12 h (Fig. 5, Supplementary Figs. 2, 3). Our previous study showed that *CzHXK1* was rapidly up-regulated 30-fold within 30 min of glucose addition to the culture^6^. Similarly, in this experiment we observed a rapid increase in *CzHXK1* mRNA in WT at 0.5 h, but not in the *hxk1* mutants (Fig. 5). Photosynthesis-related nuclear genes including *PSAH1*, *PSBO1*, *LHC16*, and *RBCS1*, which were strongly down-regulated in WT at 12 h, were insensitive to glucose in *hxk1* mutants (Fig. 5). The mitochondrial respiration gene *COX10* was slightly up-regulated in WT at 0.5 h and slightly down-regulated in WT at 12 h and minimally affected in *hxk1* at both time points (Fig. 5, Supplementary Fig. 2), suggesting that changes in respiration with glucose are not transcriptionally regulated. Likewise, glucose-responsive genes in sugar and lipid metabolism including glyceraldehyde-3-phosphate dehydrogenase (*GAP1*), fatty acid desaturase (*FAD2*), stearoyl ACP desaturase (*SAD1*), a putative sugar transporter (*SPM1*), and the major lipid droplet protein gene *MLDP1*, were up-regulated in WT but not induced in *hxk1* mutants (Fig. 5) at 12 h. Results for photosynthetic, sugar and lipid metabolism genes at 0.5 h were similar to 12 h except for *SPM1* in *hxk1-1*, which was slightly up-regulated at 0.5 h (Supplementary Fig. 2). Overall, these data provide strong evidence that CzHXK1 plays a critical role in repressing photosynthesis and up-regulating lipid metabolism with glucose.

**Fig. 6:**
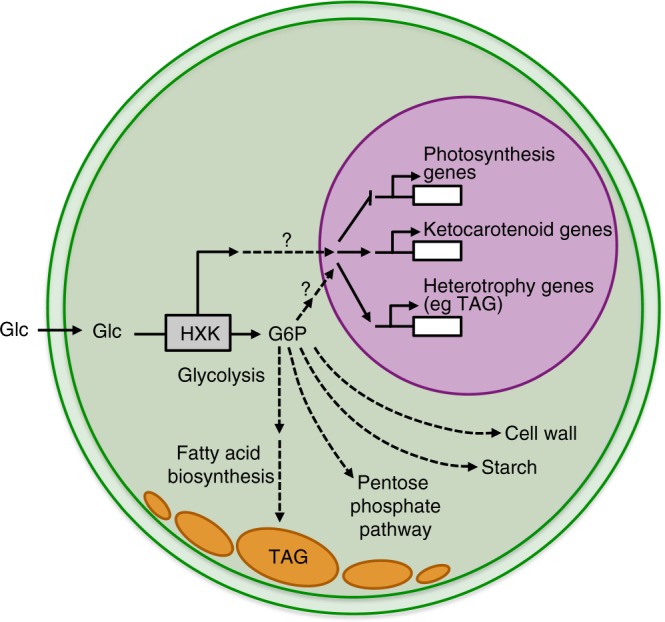
Model of hexokinase-dependent glucose pathways in *C. zofingiensis*. Hexokinase (CzHXK) catalyzes the phosphorylation of glucose (Glc) to glucose-6-phosphate (G6P) in the first step of glycolysis. G6P can be used in a variety of downstream processes, including glycolysis, starch and cell wall biosynthesis, and the pentose phosphate pathway. There are two possible mechanisms for CzHXK1-dependent glucose responses: (1) glucose-bound CzHXK1 initiates a signaling cascade with unknown intermediaries to regulate gene expression and (2) metabolic intermediates or derivatives of the glycolytic pathway are required to induce the glucose phenotype. These pathways may also work in tandem for glucose signaling and glucose responses. CzHXK-derived G6P feeds into glycolysis and fatty acid biosynthesis, resulting in the accumulation of TAGs and cytoplasmic lipid droplets

We propose a pathway of CzHXK1-dependent signaling in *C. zofingiensis* based on our physiological, microscopic, and qRT-PCR analyses of glucose responses in WT and *hxk1* mutants (Fig. 6). In the mutants, the lack of response of nuclear photosynthetic and lipid metabolism genes is consistent with the absence of changes in photosynthesis, astaxanthin and TAG accumulation, and cytoplasmic lipid droplet formation. These data suggest that HXK1 plays a direct or indirect role in repressing nuclear-encoded photosynthetic genes and activating astaxanthin and lipid accumulation. Evidence that *hxk1* mutants consume and metabolize glucose includes the increased respiration and cell volume with glucose and the growth in the dark on glucose. Therefore, these data suggest that respiration, cellular division, and cell volume growth are regulated independently from CzHXK1.

## Discussion

Sugars induce a wide range of biological changes in plants and algae that manifest at all levels of cellular activity from transcription and translation to protein stability and activity. The sugar-signaling transcriptional network impacts thousands of genes, including those involved in cell cycle, DNA and protein synthesis, amino acid metabolism, nucleotide synthesis, cell wall synthesis, glycolysis, TCA cycle, electron transport chain, carotenoid biosynthesis, fatty acid biosynthesis, protein degradation, lipid degradation, amino acid degradation, gluconeogenesis, carbohydrate metabolism, and photosynthesis^6,24–28^. In plants, there are three glucose-modulated master regulators, the glucose sensor HXK1, the energy sensor kinases KIN10/KIN11 which are inactivated by glucose, and the glucose-activated target of rapamycin (TOR) kinase^12^. Many photosynthetic organisms repress photosynthesis, decrease chlorophyll, reduce Calvin-Benson cycle enzymes, and/or accumulate starch and/or lipids in response to the preferred and most studied sugar, glucose^2,6,11,12,29–31^. Although plants and algae share many responses to glucose, there are some key differences. The most prominent difference relates to the multicellularity of plants. Sugar produced in photosynthetic tissues (source) is transported to other tissues (sink) to maintain nutrient and energy homeostasis at an organismal level^11,16^. This tissue heterogeneity makes sugar signaling complex, because plants must perceive, respond, and signal changes in different tissues, cell compartments, and developmental stages^11,16^. Multiple sugar receptors, regulators, and transduction pathways add to the complexity, leaving many questions about sugar sensing and signaling unanswered. Many algae, including *C. zofingiensis*, are unicellular, reducing the level of complexities and potentially offering new insights into sugar sensing and signaling.

In *C. zofingiensis*, glucose induces reversible changes in photosynthesis, the photosynthetic apparatus, cellular ultrastructure, lipids including thylakoid lipids and TAG, starch, astaxanthin, and gene expression pathways relating to photosynthesis, ketocarotenoid biosynthesis, metabolism, and fatty acid biosynthesis^6^. In this study, we used a forward genetics selection based on resistance to a glucose analog (2-DOG) to identify mutants that are insensitive to several glucose responses. In contrast to WT, the mutants did not (a) shut off photosynthesis, (b) accumulate high amounts of astaxanthin, TAG, or cytoplasmic lipid bodies, or (c) decrease thylakoid lipids upon addition of glucose. Photosynthesis-, metabolism-, and lipid-related genes that were differentially regulated in WT with glucose were unresponsive in glucose-treated mutants. Similar to WT, the mutants did increase cell volume and respiration, providing evidence that they did show some glucose-induced responses.

Whole-genome sequencing of the mutants revealed that CzHXK1 is necessary for the photosynthetic and metabolic switch during glucose-induced trophic transitions in *C. zofingiensis*. Intriguingly, all mutants that survived selection on 2-DOG contained a disruptive mutation in the single-copy *CzHXK1* gene at one of three locations in the gene. The fact that all the recovered mutations were in *CzHXK1* could be due to the lethality of mutations in other glucose signaling genes and/or genetic redundancy. For example, the lack of mutations in glucose transporter genes is likely due to redundancy, because *C. zofingiensis* has multiple annotated glucose transporter genes.

HXK is an evolutionarily conserved key enzyme in carbon metabolism. In the first step of the glycolytic pathway, HXK converts glucose to G6P for use in glycolysis, starch, and cell wall biosynthesis and the pentose phosphate pathway^14^. In addition to changes in cell size and respiration, the *hxk1* mutants grew in the dark with glucose, providing evidence that they are still able to metabolize glucose. *C. zofingiensis* has glucokinase^5^, which likely has lower affinity for glucose than HXK, but also phosphorylates glucose to G6P to provide a pathway for glucose metabolism. In contrast, plants lack glucokinase, however they have multiple copies of HXK.

In plants, the master regulator HXK functions as both a conserved glucose sensor and glucose-metabolizing enzyme^11,12,16^. In this study, the rapid induction of *CzHXK1* RNA abundance by glucose is consistent with the established roles of HXK in glucose metabolism, and possibly also in signaling. One challenge in understanding sugar signaling is dissociating the metabolic and signaling aspects. We present a model in which CzHXK1 plays a central role in inhibiting the transcription of nuclear-encoded photosynthetic genes, while activating genes related to heterotrophy, lipids, and ketocarotenoids resulting in the accumulation of TAGs and astaxanthin (Fig. 6). However, there are two possible mechanisms for CzHXK1-dependent changes: (1) a direct role in which glucose-bound CzHXK1 initiates a signaling cascade with unknown intermediaries that regulate nuclear gene expression, or (2) an indirect role through metabolic intermediates or derivatives of the glycolytic pathway, which are required to induce the glucose phenotype. It is possible that these two pathways work in tandem, allowing the cell to respond rapidly and robustly to changing metabolic requirement when glucose is added. This study identifies CzHXK1 as a critical molecular player required for the glucose-induced photosynthetic and metabolic switch in *C. zofingiensis*, but our data cannot distinguish whether CzHXK1 plays a signaling or metabolic role in inducing the responses. To further describe the role of HXK1 in both sensing and metabolizing glucose, it will be necessary to adapt successful efforts from yeast and plants using catalytically inactive forms of the enzyme to dissect the impact of these distinct roles in *C. zofingiensis*.

This study also suggests that the majority of accumulated TAG in glucose-grown cells begins as glucose and passes through CzHXK1 during glycolysis as it feeds into the lipid biosynthesis pathway via pyruvate. This model is consistent with our previous analysis of the glycolytic and lipid biosynthesis pathway^6^. Future work with catalytically inactive CzHXK1 will help to determine any regulatory role of CzHXK1 in TAG or astaxanthin accumulation. Because the *C. zofingiensis* genome encodes a single *CzHXK*, it provides a simple system to study HXK function, localization, interacting partners, and additional downstream signaling nodes.

In contrast to *C. zofingiensis*, the well-studied plant and yeast systems have multiple copies of *HXK*. In *Arabidopsis*, which has six HXKs, *At*HXK1 is thought to play dual roles in signaling and metabolism as well as the integration of intrinsic and extrinsic regulatory signals^11,12,16^. However, there are still unknown details regarding HXK localization and its regulatory network in plants. Mechanistic details in yeast, which has two HXKs, are better understood, and it is often used as a eukaryotic model for glucose sensing. *Sc*HXK2 shuttles in/out of the nucleus, where it stabilizes a repressor complex, preventing transcription of genes necessary to utilize carbon alternatives to glucose^31^. Although the transcriptional system and interacting players are well characterized in yeast^21^, how much of the glucose response is due to HXK signaling or catalysis is still debated^32^. Assays demonstrate that the catalytic activity of HXKs, but not their signaling activity, is interchangeable between plants and yeast^33^. Although instrumental in demonstrating the separate roles of plant HXKs, yeast is a poor model for glucose signaling in algae and plants, which tightly regulate glucose flux and gluconeogenesis in accordance with photosynthetic rates^14^. Recently, a single-copy *KnHXK1* gene was identified in the multicellular charophyte green alga *Klebsormidium nitens*, and it was shown that its signaling role is dependent on its catalytic activity when expressed in *Arabidopsis*^34^. *C. zofingiensis* is positioned at an evolutionary branch basal to land plants such as *Arabidopsis* and can be exploited as a unicellular model system for plants as well as a conceptual bridge between yeast and plants. This study shows that HXK1 is critical for sugar regulation of photosynthesis and metabolism in algae. Future studies of glucose responses at the foundation of the green lineage will improve general understanding of the critical mechanisms underpinning sugar sensing and signaling in eukaryotes.

## Methods

### Strain and growth conditions

We used the *Chromochloris zofingiensis* strain SAG 211-14 obtained from the Culture Collection of Algae at Goettingen University, whose genome was published recently^5^. The cells were grown in liquid cultures shaking at 100–150 rpm under diurnal conditions (16 h light, 8 h dark) at a light intensity of 100 μmol photons m^−2^ s^−1^ (cool white spectrum) and at 25 °C as previously described^5,6^. Cells were grown in Proteose medium (UTEX Culture Collection of Algae) with Chu’s micronutrient solution (2 mL/L, UTEX Culture Collection of Algae). For the glucose experiment, cells were grown until exponential phase (~4 × 10^6^ cells/mL) and distributed into separate beakers for acclimation 1 day prior to each experiment. For physiological measurements, the glucose addition was conducted by adding glucose to cultures at a final concentration of 35 mM, and photoautotrophic controls were maintained in parallel. Measurements were conducted after 84 h of treatment. For qRT-PCR measurements, the glucose addition experiment was conducted by adding glucose to cultures at a final concentration of 10 mM, and photoautotrophic controls were maintained in parallel. Cells were collected at 0.5 h and 12 h after the glucose addition during daylight hours. Unless otherwise specified, cells were collected by centrifugation (3200 × *g* for 5 min) and 3–4 biological replicates were used. Cells were counted and sized with the Multisizer 3 Coulter Counter (Beckman Coulter).

### Mutant generation and identification

A non-targeted, forward genetics lethal screen (selection) was used to generate *hxk1* mutants. Exponentially growing cultures (2–5 × 10^6^ cells/mL) were subjected to ultraviolet radiation (80,000 µJoules) in the Stratalinker 1800UV crosslinker (Agilent Genomics), and cells (~35,000/plate) were plated onto 5 plates with selective medium (Proteose with 5 mM 2-deoxyglucose). Cells that survived were isolated, and whole-genome sequencing was used to identify the mutations.

Genomic DNA was isolated using a cetyltrimethylammonium bromide (CTAB)-based phenol-chloroform extraction as previously described^5^. Pelleted cells were resuspended in sodium dodecyl sulfate-Edward’s Buffer (SDS-EB; 20 mM Tris/HCl, 10 mM EDTA, 100 mM NaCl, 2% SDS) and incubated with slight agitation for 2 h at 65 °C. In all, 170 µL of 5 M NaCl and 135 µL CTAB solution (274 mM CTAB, 700 mM NaCl) was added, and the solution incubated for 15 min at 65 °C. In all, 400 µL of phenol-chloroform solution (25:24:1 phenol:chloroform:isoamyl alcohol) was added, and the total mixture was vortexed before centrifugation (19,283 × *g* for 5 min). The aqueous phase was collected and incubated with 5 units RNAse A (Qiagen, Cat no. 19101) at 37 °C for 20 min. Two additional phenol-chloroform extractions were performed before adding 400 µL of 24:1 chloroform:isoamyl alcohol and vortexing. After centrifugation (19,283 × *g* for 5 min), the aqueous phase was transferred to a new tube where 0.1x volumes of 5 M NaCl and 0.7x volumes of isopropanol was added. This was mixed gently by inversion 4–6 times and centrifuged (19,283 × *g* for 15 min at 4 °C). The pellet was washed twice with 500 µL 4 °C 70% ethanol and briefly air-dried before being resuspended in 100 µL MilliQ water. DNA was incubated for 2 h at 25 °C followed by 12 h at 4 °C before quantification with a NanoDrop 2000 (Thermo Scientific).

Illumina library preparation was performed by UC Berkeley’s Functional Genomics Laboratory. Double-stranded DNA was fragmented using Covaris sonication, and size distribution was checked using an Agilent 2100 Bioanalyzer. Sequencing and barcoding adapters were added, and the size distribution was confirmed by Bioanalyzer. Illumina sequencing was performed by UC Berkeley’s Vincent J. Coates Sequencing Facility. 100 bp, paired-end reads were performed on an Illumina Hi-Seq 4000. High sequencing coverage (70–120×) was obtained for the eight mutant strains (Supplementary Table 1).

Whole-genome analysis of mutants was performed using a combination of the Broad Institute’s Genome Analysis Took Kit’s best practices^19^ and SnpEff^18^. Read quality was assessed, and low-quality reads were trimmed. FastQ reads were aligned using Burrows Wheeler Aligner^35^ using the published reference genome^5^. The output SAM files were sorted, indexed, and deduplicated using Picard Tools (GATK). GATK was used to generate a realigned BAM file from which variants were called with the Haplotype Caller tool. This naive dataset was used to bootstrap GATK’s Base Quality Score Recalibrator tool. Variants were called, extracted, and filtered on the recalibrated scores. SnpEff was used to call variants of high impact. By examining SNPs shared among the eight mutant strains and not in WT, a list of likely candidate genes was generated. Mutations in Cz*HXK1* were confirmed by Sanger sequencing. SNPs, INDELS, mutation rates, and transition/transversion rates between mutants were generated using GATK to verify that each mutant line represents an independently generated allele.

### Photosynthetic efficiency

Photosystem II efficiency was measured with the Hansatech FMS2 system as previously described^6^. Cells were dark-acclimated while shaking for 30 min. In all, 3.5 × 10^6^ cells were collected onto a glass fiber filter, which was placed into the instrument’s leaf clip. The maximum efficiency of photosystem II, (*F*_m_ – *F*_o_)/*F*_m_ = *F*_v_/*F*_m_, was measured using a 0.5 s saturating pulse (>2000 µmol quanta m^−2^ s^−1^).

### Oxygen consumption and net oxygen evolution

Oxygen consumption and net oxygen evolution were measured with the Oxygraph Plus System (Hansatech Instruments) as previously described^6^. Cells were adjusted to 1 × 10^7^ cells/mL and dark-acclimated for >30 min. Oxygen consumption was measured over 1 min after reaching steady-state in the dark at a constant temperature of 25 °C. Net oxygen evolution was also measured over 1 min after reaching steady-state under actinic light of 100 µmol photons m^−2^ s^−1^ and at a constant temperature of 25 °C. For cultures with cell volume larger than 100 µm^3^, additional samples were run with a cell density of ≤5 × 10^6^ cells/mL to achieve OD at 750 nm of ~0.2–0.4. Oxygen consumption and net oxygen evolution were normalized to cell volume, because cell volume changed during the experiment.

### Immunoblot analysis

Immunoblot analysis was conducted as previously described^6^. Cells were pelleted by centrifugation and homogenized with solubilization buffer (Tris-HCl, pH 6.8, 3.5% SDS, 6% urea, 10% glycerol) and lysing matrix D for 3 × 60 s with the FastPrep-24 5 G™ High Speed Homogenizer (6.5 m s^−1^, MP Biomedical). Chloroform-methanol protein purification was performed as previously described^36^. Proteins were resolubilized with solubilization buffer with 25 mM DTT. 10 µg of protein were run with loading buffer on Mini-PROTEAN TGX gels (Bio-Rad), transferred to PVDF membranes, and immunoblotted with anti-PSBD (1:5000; Agrisera), anti-PsbC (1,1000; Agrisera), anti-PsaA (1:1000; Agrisera), anti-PetB (1:10,000; Agrisera), anti-P17 (1:5000; from Bassi and Wollman 1991^37^), anti-LHCA2 (1:5000; Agrisera), or anti-AtpB (1:5000; Agrisera) antibodies. Because the AtpB antibody detects both chloroplast and mitochondrial F_1_β subunits, Expasy compute was used to discriminate these two variants. CzCPg01090 was predicted to be ~50 kDa and Cz03g32200 was predicted to be ~65 kDa. In addition, both PredAlgo and TargetP prediction software predicted an N-terminal mitochondrial targeting sequence for Cz03g32200. Proteins were visualized with anti-Rabbit IgG (1:10,000; GE Healthcare) and a ChemiDoc MP Imaging System (Bio-Rad). Protein concentration was determined by DC Protein Assay (Bio-Rad), and equal protein levels were confirmed by Coomassie Brillant Blue stain. Two biological replicates for each primary antibody were used.

### Pigments

Pigments were determined by high performance liquid chromatography (HPLC; 100 HPLC, Agilent) as previously described^5,6^. Cells were pelleted and homogenized with acetone and lysing matrix D for 3 × 60 s with the FastPrep-24 5 G™ High Speed Homogenizer (6.5 m s^−1^, MP Biomedical). The cell debris was pelleted by centrifugation (20,000 × *g* for 3 min) and the supernatant was collected. To ensure complete extraction, acetone extractions were repeated three times. Absorbance at wavelengths 445 nm and 520 nm were used for pigment quantification.

### Structured illumination microscopy

To stain neutral lipids, the pelleted cells were resuspended with 5 µg/mL BODIPY^TM^ 493/503 dye (Thermo Fisher Scientific) in Proteose medium as previously described^6,23^. The cells were incubated in the dark for 10 min and washed (3×) with 1 mL of Proteose medium and centrifuged (3000 × *g* for 1 min). After washing, the pelleted cells were resuspended with 50–200 µL of 0.5% low melting-point agarose prepared with Proteose medium. 4 µL of resuspended cells were immediately mounted between two microscope coverslips. After the agarose solidified, the mounted coverslips were placed in the Attofluor cell chamber (Thermo Fisher Scientific). *C. zofingiensis* live cells (technical replicates *n* ≥ 8) were observed using Zeiss Elyra PS.1 structured illumination microscopy with objective lens Plan-APOCHROMAT 100 ×/1.46 (Zeiss). Chlorophyll and BODIPY were excited by 642 and 488 nm lasers, respectively, and fluorescence from each fluorophore was acquired through 650–730 nm and 505–550 nm bandpass filters, respectively. The image acquisition was done as fully controlled by ZEN software (Zeiss). Raw images were processed to reconstruct super-resolution 3D images using ZEN software.

### Lipid extraction and thin-layer chromatography

Lipids were determined using thin-layer chromotography (TLC). In all, 3.3 × 10^6^ µm^3^ of cells were flash frozen in liquid N_2_, and samples were normalized to culture biomass using the Multisizer 3 Coulter Counter (Beckman Coulter). Frozen pellets were broken in an MP Biosciences MP-24 bead beater using lysing matrix D in a 2-mL screw-capped tube (6.5 m/s, 60 s, 3 times, −20 °C). In all, 1 mL of 2:1 chloroform:methanol was added to the disrupted cells and placed on a vortex for 5 min. 266 µL of 0.73% (w/v) NaCl solution was added, and the mixture was inverted 5-6 times to mix. Samples were then centrifuged for 5 min at 10,000 × *g* (9,000 RCF). The lower, solvent phase was removed. In all, 200 µL of this total lipid extract was dried under an N_2_ stream and resuspended to 33.3 µL in chloroform. In total, 10 µL of the concentrated lipid extract was loaded onto a clean silica TLC plate. These were developed in either acetone:toluene:water (91:30:8) or hexane:diethyl ether:glacial acetic acid (91:39:1.3). Lipids were visualized by sulfuric acid spray and charring (25% H_2_SO_4_ in 50% ethanol, 100 °C for 6 min). Standards are as follows: Oleic acid (*cis*-9-Octadecenoic acid, Sigma Aldrich catalog number O1008, 125 µg loaded), TAG (Glyceryl trilinoleate, Sigma Aldrich catalog number T9517, 50 µg loaded), MGDG (monogalactosyldiacylglycerol, Avanti Polar Lipids catalog number 840523, 0.50 µg loaded), DGDG (digalactosyldiacylglycerol, Avanti Polar Lipids catalog number 850524, 40 µg loaded). Store-bought olive oil was extracted as described above, and 2.5 µL of extract was loaded. TLC was conducted for two biological replicates of each treatment.

### qRT-PCR

RNA was extracted from WT, *hxk1-1*, and *hxk1-2* cells as described^5,6,38^ with the modification of increased DNAse treatment time to 60 min. A cDNA library was generated using the OmniScript RT Kit (Qiagen) with slight modifications to the manufacturer’s instructions. Two microgram RNA template was added to a total reaction volume of 20 µL. Both random nonamers (15n, 10 µM final concentration) and oligo dT (15 T, 1 µM final concentration) were used to ensure efficient reverse transcription without biasing towards polyadenylated transcripts. Four units of Omni RT enzyme were added as well as 10 units of RiboLock RNAse inhibitor and mixed by inversion. Reactions were briefly spun down and then incubated at 37 °C for 2 h. Negative RT control was performed identically by adding water in place of the Omni RT enzyme. qRT-PCR primers were designed with a *T*_m_ of 60 °C using Primer3 (Supplementary Table 2). qRT-PCR primers were designed to span exon-exon junctions and were biased towards the 3′ end of the transcript where possible. Primer sets were optimized to have a doubling efficiency between 95–101%. qRT-PCR was performed using SybrGreen according to the manufacturer’s recommendations on an Applied Biosystems 7500 instrument. In all, 5 µL of 1/5x RT reaction was used as a template. qRT-PCR expression level was quantified using the ΔΔC_T_ method^39^. Cz04g37020 (Aspartate-prephenate aminotransferase) was used as an internal reference gene due to its robust FPKM level and low variability in RNA-Seq data^6^.

### Statistics and reproducibility

Data are presented as means ± SD (*n* = 3–4) with individual data points shown for photosynthetic efficiency, oxygen consumption, net oxygen evolution, and pigments. Immunoblots and thin-layer-chromatography from two biological replicates were conducted. For structured illumination microscopy, technical replicates (*n* *≥* 8) were observed. For qRT-PCR, data represent means ± SD of three biological replicates consisting of three technical replicates each.

### Reporting summary

Further information on research design is available in the Nature Research Reporting Summary linked to this article.

## Supplementary information

Supplementary Information

Description of Additional Supplementary Files

Reporting Summary

Peer Review File

Supplementary Data 1

## Data Availability

Whole-genome sequencing data have been deposited in the NCBI Sequence Read Archive with the primary accession code SUB5868024. Raw data used to generate plots can be found in Supplementary Data 1. All data and algal material that support the findings of this study are available from the corresponding authors (M.S.R. and K.K.N.) upon reasonable request.
