# Supplementary material for: Hexokinase is necessary for glucose-mediated photosynthesis repression and lipid accumulation in a green alga
Source: Commun Biol. 2019 Sep 19;2:347. doi: 10.1038/s42003-019-0577-1 (PMC6753101; doi:10.1038/s42003-019-0577-1)
Supplement: Supplementary file 1 — Supplementary Information [file 42003_2019_577_MOESM1_ESM.pdf]

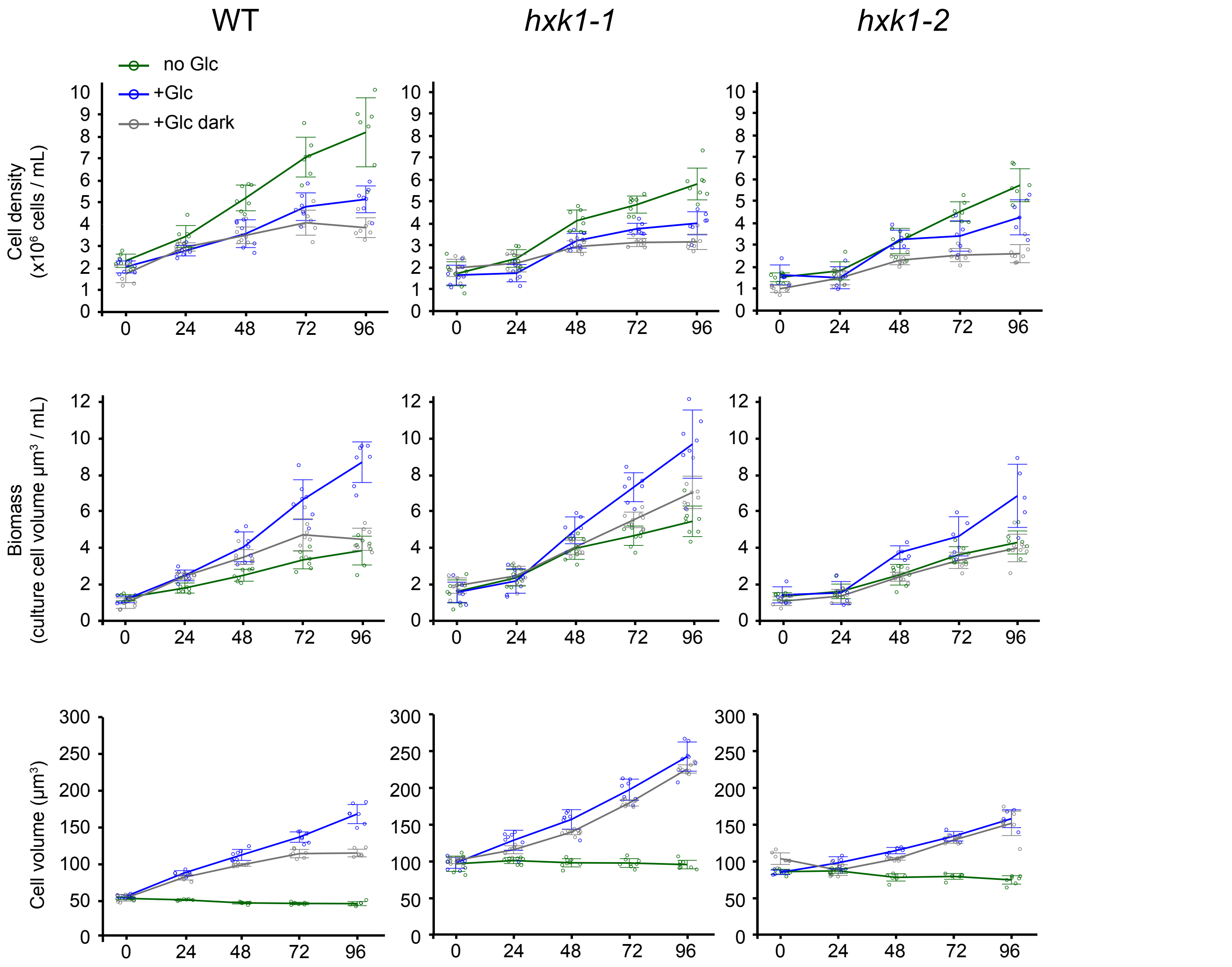

Supplementary Figure 1. Growth of WT and *hxk1* mutants under various conditions. Cell density, culture biomass, and individual cell volume of cells grown without glucose in the light (green), with glucose in the light (blue), and with glucose in the dark (grey). Data represent means ± SD (n = 5-8 biological replicates, individual data points shown).

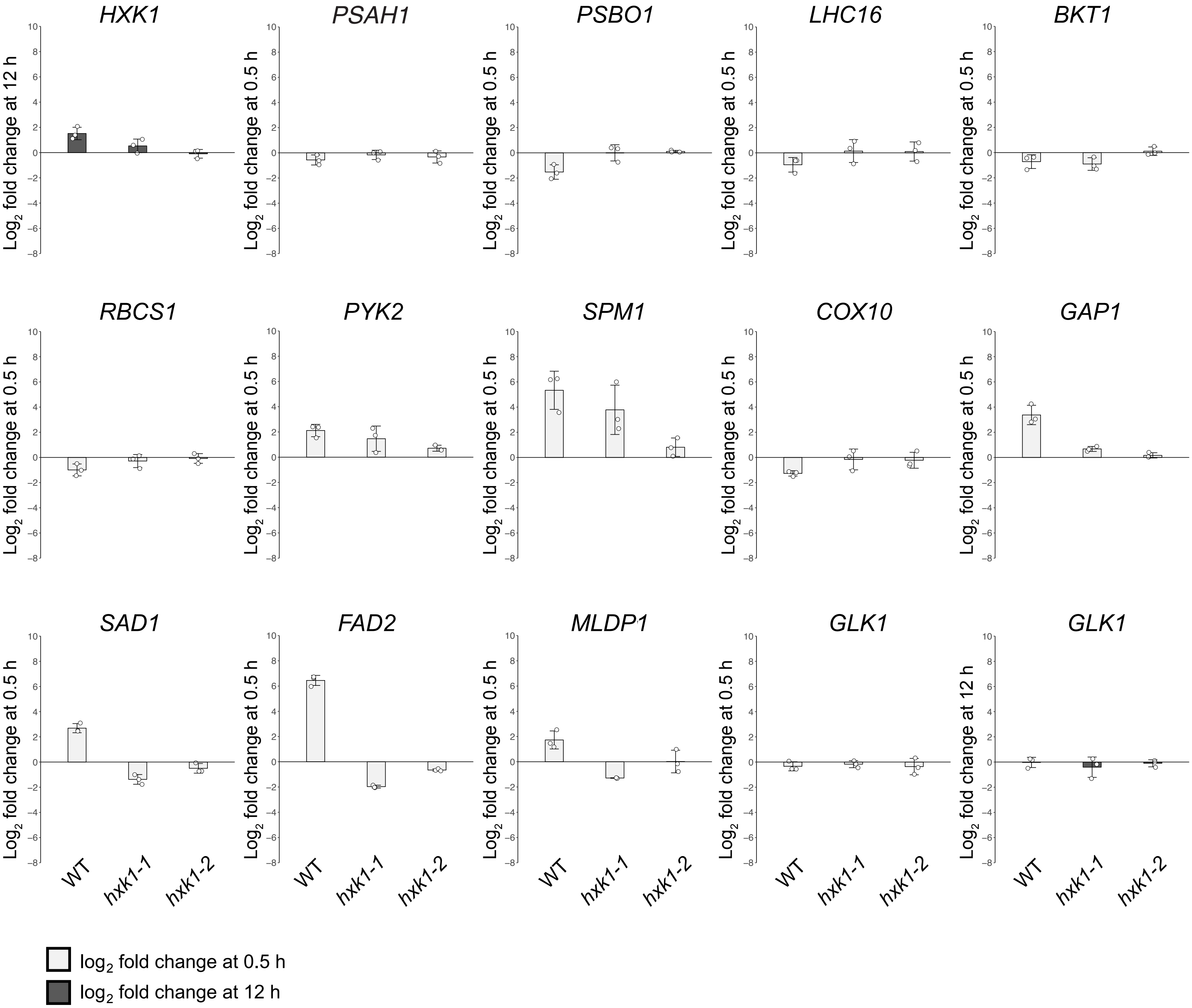

Supplementary Figure 2. The transcriptional response of *hxk1* mutants to glucose is attenuated. qRT-PCR analysis of mRNA levels of select photosynthetic and metabolic genes (identified by RNA-Seq[6]) in WT and *hxk1* mutants. The log_2-transformed fold change of mRNA level with glucose relative to a time-matched control without glucose at 12 h for *HXK1* and *GLK1*, and 0.5 h for all other plots. Additional time points are shown in Fig. 5. Data represent means ± SD (n = 3 biological replicates, individual data points shown). Raw ΔC_T data and biological replicates are shown in Supplementary Fig. 3.

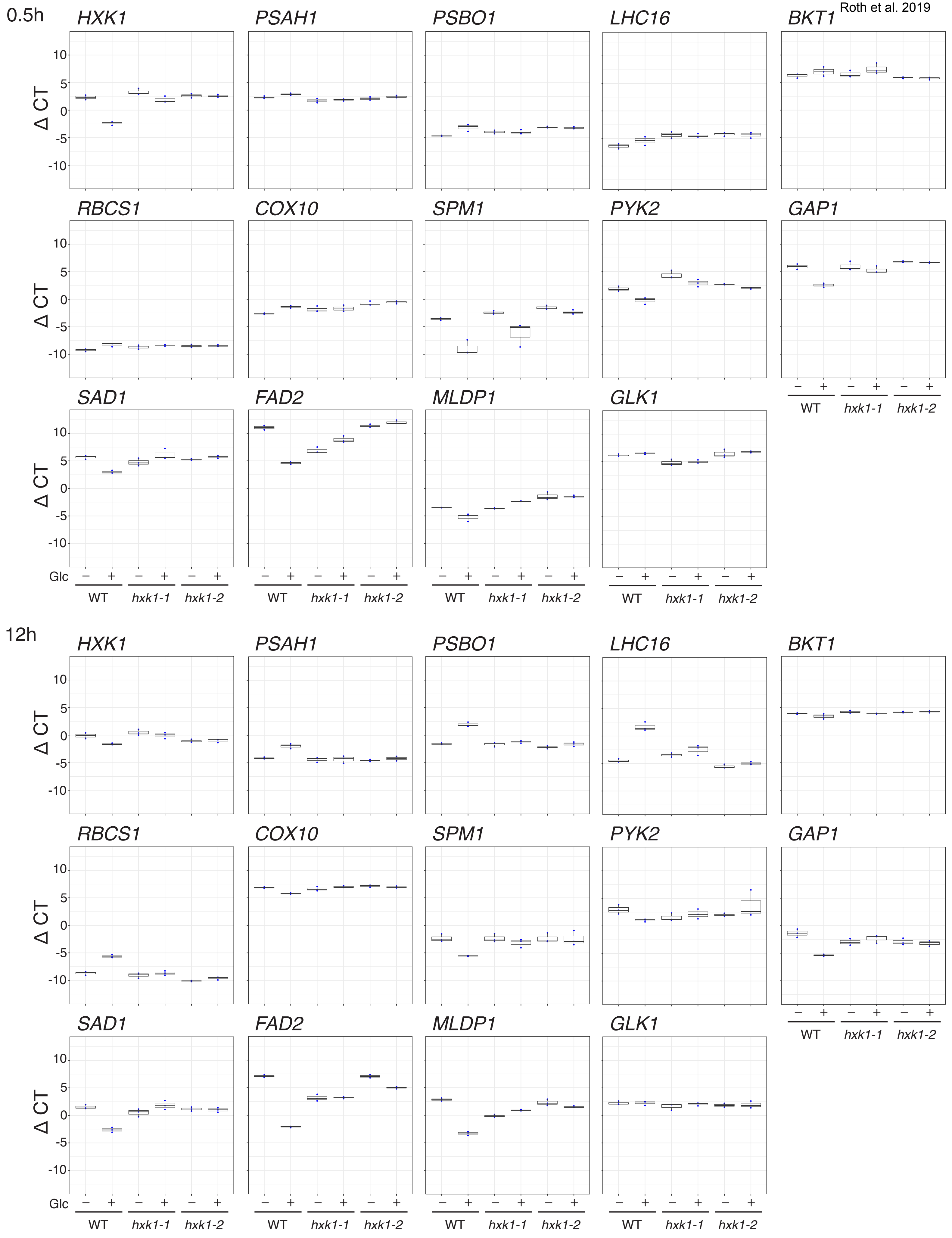
Roth et al. 2019
0.5h
HXK1
PSAH1
PSBO1
LHC16
BKT1
RBCS1
COX10
SPM1
PYK2
GAP1
SAD1
FAD2
MLDP1
GLK1
Δ CT
10
5
0
-5
-10
Glc
− + − + − +
WT
hxk1-1
hxk1-2
12h
HXK1
PSAH1
PSBO1
LHC16
BKT1
RBCS1
COX10
SPM1
PYK2
GAP1
SAD1
FAD2
MLDP1
GLK1
Δ CT
Glc
WT
hxk1-1
hxk1-2

Supplementary Figure 3. Raw qRT-PCR data. qRT-PCR raw ΔC_T (see Methods) of biological triplicates at 0.5 h and 12 h with (+) and without (–) glucose. Box is centered on the mean; the thick line represents the median; the bottom of the box represents the 25th percentile; the top of the box represents the 75th percentile; upper and lower whiskers are ± 1.5 interquartile range; and dots represent biological triplicates.

AtpB (membrane cut at 37 kDa, bottom used for LHCII)

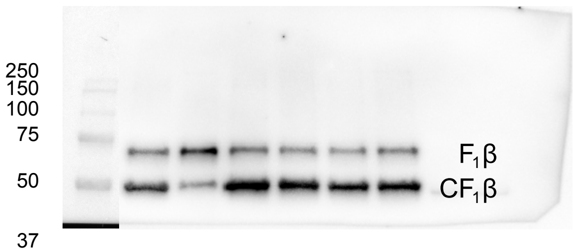

LHCII (membrane cut at 37 kDa, top used for AtpB)

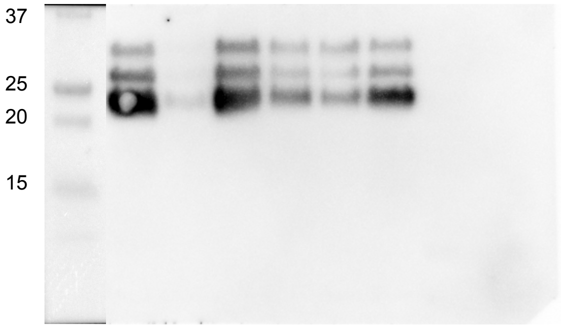

CP43 (membrane cut above 25 kDa, bottom used for Cyt b_6)

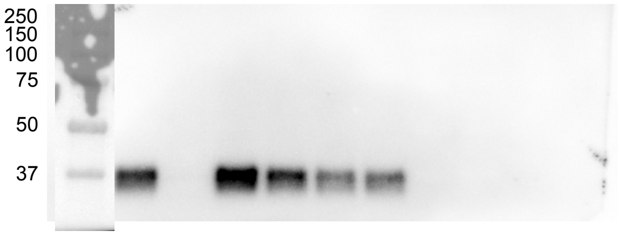

Cyt b_6 (membrane cut above 25 kDa, top used for CP43)

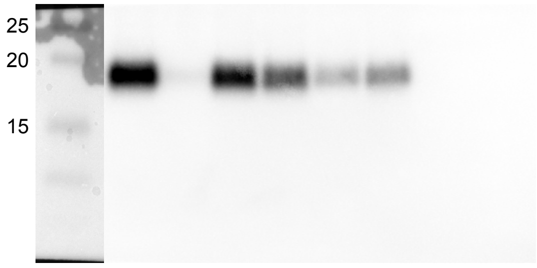

PsaA (membrane cut at 37 kDa, bottom used for D2)

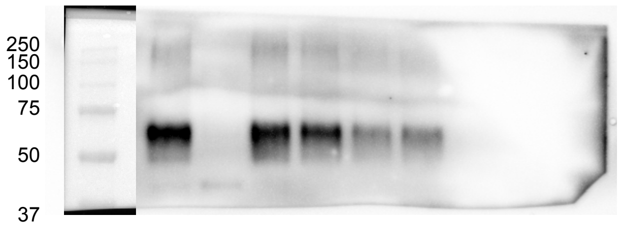

D2 (membrane cut at 37 kDa, top used for PsaA)

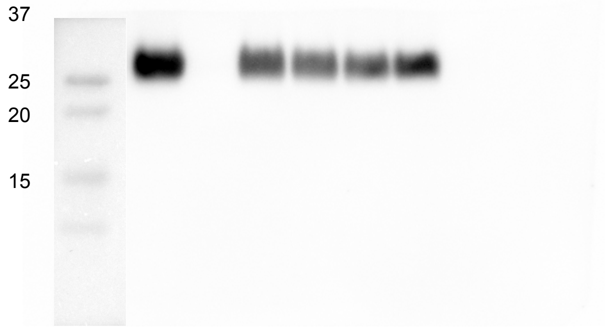

LHCI (membrane cut at 37 kDa, no ladder image taken)

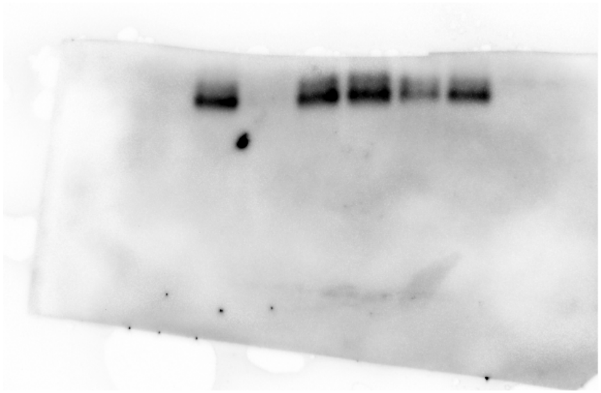

CBB gel (both sets of replicates)

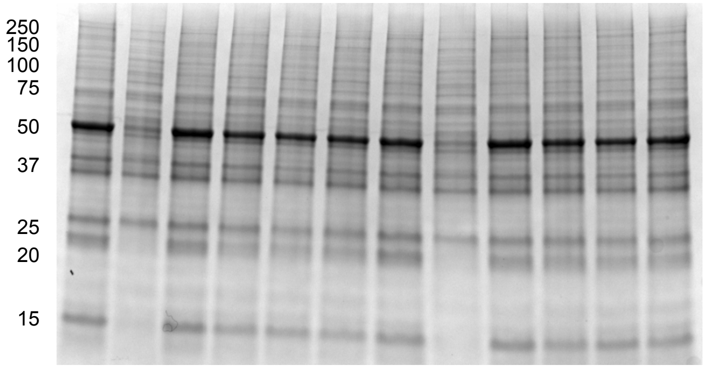

Supplementary Figure 4. Supplementary material for immunoblots in Figure 1D. Immunoblot methods including antibody concentrations are described in Methods. Samples were normalized to total protein, and 10 µg of protein were loaded in each well. After protein transfer, the membranes were cut to use with antibodies of different sizes. Unspecific immunoreactions of incorrect size were observed with the PsaA antibody. The protein marker (Bio-Rad, Precision Plus Protein™ All Blue Prestained Protein Standards #1610373) was used as a reference and imaged with white illumination.

Supplementary Table 1. Summary of *hxk1* mutants

Mutant Strain	Class	Mutation	Type	Predicted Result	Coverage (x)
hxk1-1	1	C471CG	Insertion	Early Stop Codon Exon 2	*116.4*
hxk1-2	1	C471CG	Insertion	Early Stop Codon Exon 2	*85.8*
hxk1-3	2	C3426T	missense	Early Stop Codon Exon 7	92.4
hxk1-4	2	C3426T	missense	Early Stop Codon Exon 7	94.9
hxk1-5	3	T4795G	missense	Missplice	69.1
hxk1-6	3	T4795G	missense	Missplice	92.6
hxk1-7	3	T4795G	missense	Missplice	80.9
hxk1-8	3	T4795G	missense	Missplice	102.3
**Sum**					
**Average**					91.8

continue Supplementary Table 1

Mutant Strain	Total Number of Variants	Number of Potential Effects	Variant Rate
hxk1-1	717	2835	1.23E-05
hxk1-2	625	2517	1.08E-05
hxk1-3	622	2511	1.07E-05
hxk1-4	663	2686	1.14E-05
hxk1-5	634	2618	1.09E-05
hxk1-6	778	3028	1.33E-05
hxk1-7	646	2514	1.11E-05
hxk1-8	733	2922	1.26E-05
**Sum**	5418	21631	9.30E-05
**Average**	677.3	2703.9	0.0

continue Supplementary Table 1

Mutant Strain	SNPs	Insertions	Deletions	Transitions	Transversion	Ts/Tv Ratio
hxk1-1	497	105	115	323	174	1.856
hxk1-2	400	99	126	238	162	1.469
hxk1-3	366	119	137	213	153	1.392
hxk1-4	412	111	140	240	172	1.395
hxk1-5	434	78	122	268	166	1.614
hxk1-6	555	106	117	340	215	1.581
hxk1-7	441	89	116	275	166	1.657
hxk1-8	499	106	128	322	177	1.819
**Sum**	3604	813	1001	2219	1385	1.602
**Average**	450.5	101.6	125.1	277.4	173.1	1.6

continue Supplementary Table 1

Mutant Strain	A>C	A>G	A>T	C>A	C>G	C>T	G>A	G>C	G>T	T>A	T>C	T>G
hxk1-1	16	67	11	16	32	78	121	21	25	25	57	28
hxk1-2	12	61	24	14	25	56	66	26	66	15	55	20
hxk1-3	21	59	11	16	29	46	60	24	12	18	48	22
hxk1-4	25	63	16	18	25	62	57	22	24	18	58	24
hxk1-5	21	59	15	24	24	69	77	23	19	15	63	25
hxk1-6	22	65	29	27	29	102	105	25	27	18	68	28
hxk1-7	21	54	14	22	32	74	81	18	12	27	66	20
hxk1-8	23	78	22	21	26	85	90	19	13	25	69	28
**Sum**	161	506	142	158	222	572	657	178	198	161	484	195
**Average**	20.1	63.3	17.8	19.8	27.8	71.5	82.1	22.3	24.8	20.1	60.5	24.4

Supplementary Table 2. Primers used for qRT-PCR analyses

Gene ID (v5.2.3.2)	Gene name	Forward	Reverse
Cz13g07170	*HXK1*	CCGTGTGGTTTATGTGAAGCTG	TCGGCCGGTGTATTTGTCAATA
Cz12g12230	*PSAH1*	AAGGTCTGCGCAAAGTATGGCG	GGTTCTGCAGGGGGTTGTAACG
Cz16g19040	*PSBO1*	AGGAGAACAACAAGAGCGCA	ATGGACTCGAATACACCGGC
Cz13g03240	*LHC16*	AATGAGAACCTGGTGCACGCAC	CAACCTGTCCAGACCCTCACCA
Cz13g13100	*BKT1*	CCGTGTGGTTTATGTGAAGCTG	TCGGCCGGTGTATTTGTCAATA
Cz17g13100	*RBCS1*	GCCTACACTGCCCAGTACAA	TTGGCAGCTTCCACATAGTC
Cz02g32130	*COX10*	TGCCTTGTTCTTCTGGCAGATG	CTATGTTTGACAGCATGCGGAAG
Cz07g18160	*SPM1*	CTTTCGTCATTGGCCAGTGC	GAAGAGCTGCGCAATGAACA
Cz01g21060	*PYK2*	CCGCCATCACTTTGACTTCC	GACAGGCTGCCATTCTTTGT
Cz05g34160	*GAP1*	GGCACCGTAGAAGGCACAAAGG	CATGGGATCTTGGTGGGGTCCA
Cz04g09090	*SAD1*	AAAGACAGCTGTCCATAGGGTG	GTCCATTGCATCAGATGAGGGA
Cz03g33220	*FAD2*	TGTTCAGTCAGCTGCCACATTA	GCAGATGTTGGTTTCTTGCCAT
Cz04g29220	*MLDP1*	CATCCTTTGGACCTGTCTCGAA	CAGGAAGGGACACGATGGAATT
Cz06g03010	*GLK1*	CATGTTCCTGGCCATTGTGGG	ACGATCCAGCAACCGAGGCATA
Cz04g37020	*APPS1*	CAGGGCTTTGGGTCGGTG	ACCAAATGCATCCCCGGG
