# Supplementary material for: Hexokinase is necessary for glucose-mediated photosynthesis repression and lipid accumulation in a green alga
Source: Commun Biol. 2019 Sep 19;2:347. doi: 10.1038/s42003-019-0577-1 (PMC6753101; doi:10.1038/s42003-019-0577-1)
Supplement: Supplementary file 2 — Description of Additional Supplementary Files [file 42003_2019_577_MOESM2_ESM.docx]

Description of Additional Supplementary Files

File Name: Supplementary Data 1

Description: Raw data used to generate plots.
