# Supplementary material for: Hexokinase is necessary for glucose-mediated photosynthesis repression and lipid accumulation in a green alga
Source: Commun Biol. 2019 Sep 19;2:347. doi: 10.1038/s42003-019-0577-1 (PMC6753101; doi:10.1038/s42003-019-0577-1)
Supplement: Supplementary file 3 — Reporting Summary [file 42003_2019_577_MOESM3_ESM.pdf]

natureresearch

Corresponding author(s): Melissa Roth and Krishna Niyogi

Reporting Summary

Nature Research wishes to improve the reproducibility of the work that we publish. This form provides structure for consistency and transparency in reporting. For further information on Nature Research policies, see Authors & Referees and the Editorial Policy Checklist.

Statistical parameters

When statistical analyses are reported, confirm that the following items are present in the relevant location (e.g. figure legend, table legend, main text, or Methods section).

n/a	Confirmed	
☐	☒	The exact sample size (*n*) for each experimental group/condition, given as a discrete number and unit of measurement
☐	☒	An indication of whether measurements were taken from distinct samples or whether the same sample was measured repeatedly
☒	☐	The statistical test(s) used AND whether they are one- or two-sided<br>*Only common tests should be described solely by name; describe more complex techniques in the Methods section.*
☒	☐	A description of all covariates tested
☒	☐	A description of any assumptions or corrections, such as tests of normality and adjustment for multiple comparisons
☐	☒	A full description of the statistics including central tendency (e.g. means) or other basic estimates (e.g. regression coefficient) AND variation (e.g. standard deviation) or associated estimates of uncertainty (e.g. confidence intervals)
☒	☐	For null hypothesis testing, the test statistic (e.g. *F*, *t*, *r*) with confidence intervals, effect sizes, degrees of freedom and *P* value noted<br>*Give P values as exact values whenever suitable.*
☒	☐	For Bayesian analysis, information on the choice of priors and Markov chain Monte Carlo settings
☒	☐	For hierarchical and complex designs, identification of the appropriate level for tests and full reporting of outcomes
☒	☐	Estimates of effect sizes (e.g. Cohen's *d*, Pearson's *r*), indicating how they were calculated
☐	☒	Clearly defined error bars<br>*State explicitly what error bars represent (e.g. SD, SE, CI)*

Our web collection on statistics for biologists may be useful.

Software and code

Policy information about availability of computer code

Data collection	ZEN software (Zeiss Microscopy v2012 SP5) was used to acquire and reconstruct images for SIM.
Data analysis	Adaptor sequences were trimmed using Sickle (Version 1.33). Read quality was assessed using FastQC (Version 0.11.2). FastQ reads were aligned using Burrows Wheeler Aligner (Version 0.7.12-r1039). Picard Tools (Version 1.138) was used to process SAM and BAM files. Samtools (Version 1.5) was used to index SAM files. The Genome Analysis Tool Kit (GATK Version 3.4.46) was used to identify and filter SNPs, Insertions and Deletions. The impact of those mutations were assessed using SnpEff (Version 4.3p). Specific mutations, mutation rates, and Ts/Tv ratios were calculated using VCFtools (Version 0.1.13).

For manuscripts utilizing custom algorithms or software that are central to the research but not yet described in published literature, software must be made available to editors/reviewers upon request. We strongly encourage code deposition in a community repository (e.g. GitHub). See the Nature Research guidelines for submitting code & software for further information.

Data

Policy information about availability of data

All manuscripts must include a data availability statement. This statement should provide the following information, where applicable:

- Accession codes, unique identifiers, or web links for publicly available datasets
- A list of figures that have associated raw data
- A description of any restrictions on data availability

Whole genome sequencing data have been deposited in the NCBI Sequence Read Archive with the primary accession code SUB5868024. Raw data used to generate plots can be found in Supplementary Data 1. All data and algal material that support the findings of this study are available from the corresponding authors (M.S.R. and K.K.N.) upon reasonable request.

Field-specific reporting

Please select the best fit for your research. If you are not sure, read the appropriate sections before making your selection.

☒ Life sciences ☐ Behavioural & social sciences ☐ Ecological, evolutionary & environmental sciences

For a reference copy of the document with all sections, see nature.com/authors/policies/ReportingSummary-flat.pdf

Life sciences study design

All studies must disclose on these points even when the disclosure is negative.

Sample size	Samples size was assessed through preliminary experiments. Replicates of at least 3-4 were used in this study and are standard for the field.
Data exclusions	No data were excluded in this study.
Replication	All the data we presented in this study were successfully reproduced.
Randomization	Samples were randomly allocated into experimental groups.
Blinding	Blinding was not used and would have no effect on the outcome of data output from instruments or analyses.

Reporting for specific materials, systems and methods

Materials & experimental systems

n/a	Involved in the study	
☐	☒	Unique biological materials
☐	☒	Antibodies
☒	☐	Eukaryotic cell lines
☒	☐	Palaeontology
☒	☐	Animals and other organisms
☒	☐	Human research participants

Methods

n/a	Involved in the study	
☒	☐	ChIP-seq
☒	☐	Flow cytometry
☒	☐	MRI-based neuroimaging

Unique biological materials

Policy information about availability of materials

Obtaining unique materials	All unique materials used are readily available from the authors.

Antibodies

Antibodies used	Antibodies used in this study were Anti-PsbD (D2) global antibody (Agrisera AS06 146, 1/5000 dilution), Anti-PsbC (CP43) antibody (Agrisera AS11 1787, 1/3000), Anti-PsaA antibody (Agrisera AS06 172, 1/1000 dilution), Anti-Lhca2 antibody (Agrisera AS01 006, 1/5000 dilution), Anti-AtpB global antibody (Agrisera AS05 085, 1/5000 dilution), Anti-P17 antibody (Bassi and Wollman, 1991, 1/5000 dilution) and anti-Rabbit IgG, HRP-linked whole antibody (GE Healthcare Life Sciences NA9341ML).

Validation

All primary antibodies were validated by Agrisera (www.agrisera.com) for closely related green algal species, except for P17 which was validated in a closely related green algal species by Bassi and Wolleman 1991. In this study, all antibodies were bands of the expected size and as shown in Figure 1D , except for PsaA in which we selected the appropriately sized and darkest band (full blot shown in Supplementary Data 1).
