# Supplementary material for: Hexokinase is necessary for glucose-mediated photosynthesis repression and lipid accumulation in a green alga
Source: Commun Biol. 2019 Sep 19;2:347. doi: 10.1038/s42003-019-0577-1 (PMC6753101; doi:10.1038/s42003-019-0577-1)
Supplement: Supplementary file 4 — Peer Review File [file 42003_2019_577_MOESM4_ESM.pdf]

Editorial Note: This manuscript has been previously reviewed at another journal. This document only contains reviewer comments and rebuttal letters for versions considered at *Communications Biology* .

REVIEWERS' COMMENTS:

Reviewer #1 (Remarks to the Author):

The authors have carefully addressed most of the comments raised by the reviewers. They have adequately updated the text and performed useful additional experiments.

The revised manuscript is therefore strongly improved.

Stephane Lemaire
